# Changes of gluteus medius muscle in the adult patients with unilateral developmental dysplasia of the hip

**DOI:** 10.1186/1471-2474-13-101

**Published:** 2012-06-15

**Authors:** RuiYu Liu, XiaoDong Wen, ZhiQin Tong, KunZheng Wang, ChunSheng Wang

**Affiliations:** 1 Department of Orthopedic Surgery, Second Hospital Affiliated to the Medical College of Xi’an Jiaotong University, Xi’an Shaanxi, 710004, People's Republic of China; 2 Department of Radiology, Second Hospital Affiliated to the Medical College of Xi’an Jiaotong University, Xi’an Shaanxi, 710004, People's Republic of China

## Abstract

**Background:**

The gluteus medius muscle is essential for gait and hip stability. Changes that occur in the gluteus medius muscles in patients with developmental dysplasia of the hip (DDH) are not well understood. A better understanding of DDH related changes will have positive repercussions toward hip soft tissue reconstruction.

**Methods:**

19 adult patients with unilateral DDH scheduled for total hip arthroplasty were assessed for: cross-sectional area (CSA), radiological density (RD) and the length of gluteus medius using computed tomograhpy(CT) (scanned before THA). Hip abductor moment arm and gluteus medius activation angle were also measured *via* hip anteroposterior radiographs.

**Results:**

Both CSA and RD of gluteus medius muscle were significantly reduced (p < 0.05) in the affected hip compared to the control. In the affected hip, the length of the gluteus medius muscle was reduced by 8-11 % (p < 0.05) while the gluteus medius activation angle was significantly increased (p < 0.05) and the hip abductor moment arm was decreased (p < 0.05).

**Conclusions:**

The gluteus medius showed substantial loss of CSA, RD as well as decreased length in patients with DDH in the affected hip. These changes should be considered in both hip reconstruction and postoperative rehabilitation training in patients with DDH.

## Background

Total hip arthroplasty performed on patients with hip dysplasia presents challenging work for surgeons due to its bony deformity and soft tissue imbalance. The bony configuration changes of hip dysplasia have been investigated extensively, however, soft tissue abnormalities around the affected hip have been less well studied. Such characterization is essential to help recognize hip dysplasia and to make an optimal plan for hip reconstruction and postoperative rehabilitation in patients with developmental dysplasia of the hip (DDH) [1,2].

Abduction muscle tension is important for gait and hip biomechanics. Abductor dysfunction may lead to joint instability, resulting in a high rate of recurrent dislocation [3,4]. The gluteus medius muscle, which plays a key role in exerting abduction force across the hip joint, providing stabilization of the pelvis during single leg stance [5], is the main muscle in the hip abductor muscle group that also includes gluteus maximus, gluteus minimus, piriformis muscles, and tensor fascia lata. Insufficiency of the gluteus medius muscle is clinically associated with a positive Trendelenburg sign and finally leads to a loss of pelvic control with an impaired ability to walk. The preservation of the gluteus medius muscle during total hip arthroplasty is therefore regarded to be crucial for postoperative abduction force [6] and functional outcome.

With the femur displaced proximally, the gluteus medius muscle and other soft tissues around the affected hip contracts in patients with DDH and leads to leg length discrepancy and hip instability [7]. It has been suggested that the gluteus medius muscle as well as other soft tissues be released to obtain femoral reduction [8,9] and optimal biomechanics in patients with DDH during total hip arthroplasty. Previous studies [10,11] compared length changes of the gluteus medius muscle between the pelvic support osteotomy preoperative and posteroperative in the congenital dislocation of the hip. However, fewer studies quantified the contracture extent (the length change and atrophy) of gluteus medius muscle compared to the healthy hip in patients with DDH, which is vital for femoral reduction and abductor muscle strength.

Abductor weakness often can not be corrected in patients with DDH performed THA postoperatively [12-14]. Abductor strength is related not only with abductor muscle strength itself but also other factors: Abduction strength correlated positively with both femoral offset and the length of the abductor lever arm. Thus, both the gluteus medius muscle itself and related factors should be explored to better understand DDH and possible recovery strategies. These specific data may be contributive to helping us understand the abnormalities of the gluteus medius and in making optimal abductor function reconstruction, in addition to the design of more specifically helpful rehabilitation training programs.

Computed tomography has been used to assess the muscle change in the trunk [15] and around the hip [16-18]. Cross-sectional area (CSA) is closely related to the muscle volume and the radiological density of muscle, which indirectly represents muscle strength. The purpose of our study is to better characterize the gluteus medius muscle abnormalities in patients with adult unilateral DDH. Our approach to achieve this goal was to: 1) quantify the loss of contractile muscle mass in the gluteus medius muscle (responsible for hip abduction strength) and 2) investigate the length changes of the gluteus medius muscle (knowledge of which is essential for femur reduction in hip reconstruction in patients with DDH).

## Methods

### Patients

We reviewed the CT scans of 19 unilateral dysplasia hips collected recently over the course of three years. The scans were made for morphological evaluation of the acetabular and proximal femur before total hip arthroplasty [19]. The overall average age of the patients was 47 years (range 35–61 years), the mean weight was 55 kg (range 50–69 kg), and the mean height was 157 cm (range 155–170 cm). There were no significant difference in age, weight, or height between the two groups (7 males and 12 females).

According to the classification of Crowe [20], there were 8 hips in class II and 11 hips in class III in our patients with unilateral developmental dysplasia of the hip. The Crowe classification divides dysplastic hips radiographically into four categories based on the extent of proximal migration of the femoral head. Class I is less than 50 per cent subluxation; class II is 50 to 75 per cent subluxation; class III is 75 to 100 per cent subluxation; and class IV, more than 100 per cent subluxation. The degree of hip subluxation ranged from 55 to 98 percent according to the method of Crowe [20]. None of the patients had accepted osteotomy for treatment or correction of their hip disease. Medical history, duration of hip symptoms and the use of medications for pain relief were noted. Patients were instructed to maintain their normal medication on all test occasions. Patients gave written consent and the Ethical Committee of our institution approved the study.

### Computed tomography

The patients were examined in the supine position in a Phillips MX 8000 (Phillips Medical Systems, Cleveland, Ohio, USA) spiral CT-scanner with the pelvis in the neutral position the lower limb placed in internal rotation so the patella is situated in the frontal plane. Routine radiographs of the anteroposterior pelvis were taken. Transverse images were obtained of each patients using the following CT protocol. The area from the iliac crest to below the lesser trochanter was scanned, 2.0 mm thick slices at 2.0 mm intervals. The scanned images were DICOM format(512 × 512 pixels) and were analyzed *via* computerized tomography work station.

**Figure 1:**
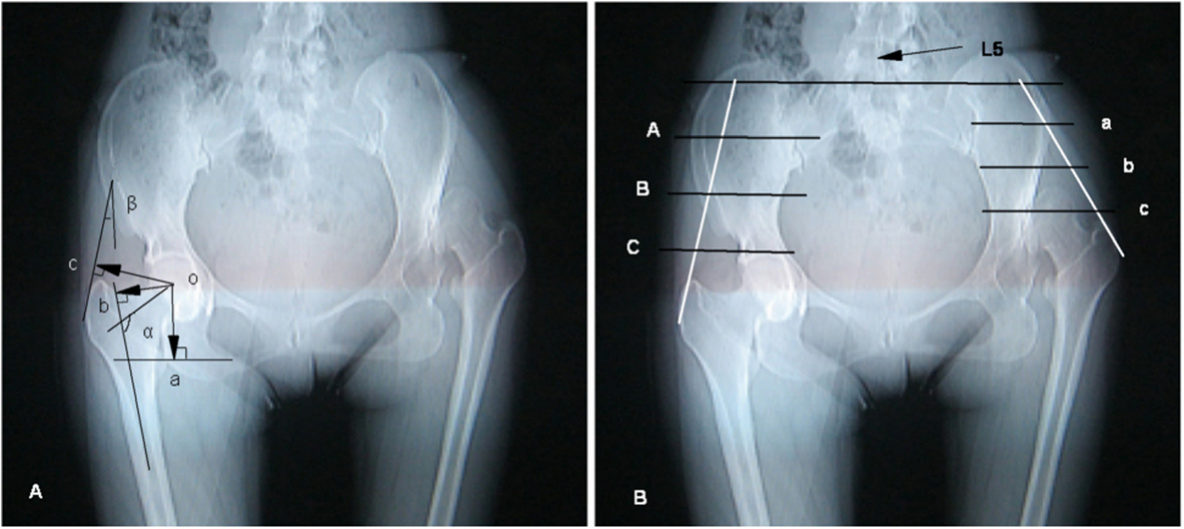
**Diagram of Measurement parameters and scanning planes on pelvic radiographs. A** Depicting the different lines and angles measured on pelvic scout view. “o” is the center of femoral head; line “oa” is height of the center of femoral head; line “ob” is femoral offset; line “oc” is the abductor muscle lever arm; angle α is neck-shaft angle. **B** Illustration of the three anatomical levels at which the gluteus medius muscle was measured. **A-C** planes (red line) are on the healthy side and **a-c** planes are on the affected sides.

Definitions of observed parameters: The femoral offset (FO) was the distance from the center of rotation of the femoral head to a line vertical to the long axis of the femur [21]. The height of the center of the femoral head was the distance from the center of rotation of the femoral head to a line vertical to horizontal line through the midpoint of the lesser trochanter. The neck-shaft angle was measured as the angle between the midline of the femoral neck and the midline of the femoral shaft [22]. The abductor lever arm was determined as the perpendicular distance from a line tangential to the greater trochanter to the center of rotation of the femoral head. The tangential line corresponded to the abductor shadow [23], and the gluteus medius activation angle was delimited by the line tangential to the greater trochanter and the line running from the insertion point of the gluteus medius to the global offset laterally [24]. These parameters were measured on a magnified scout view of CT (shown in Figure 1A).

**Figure 2:**
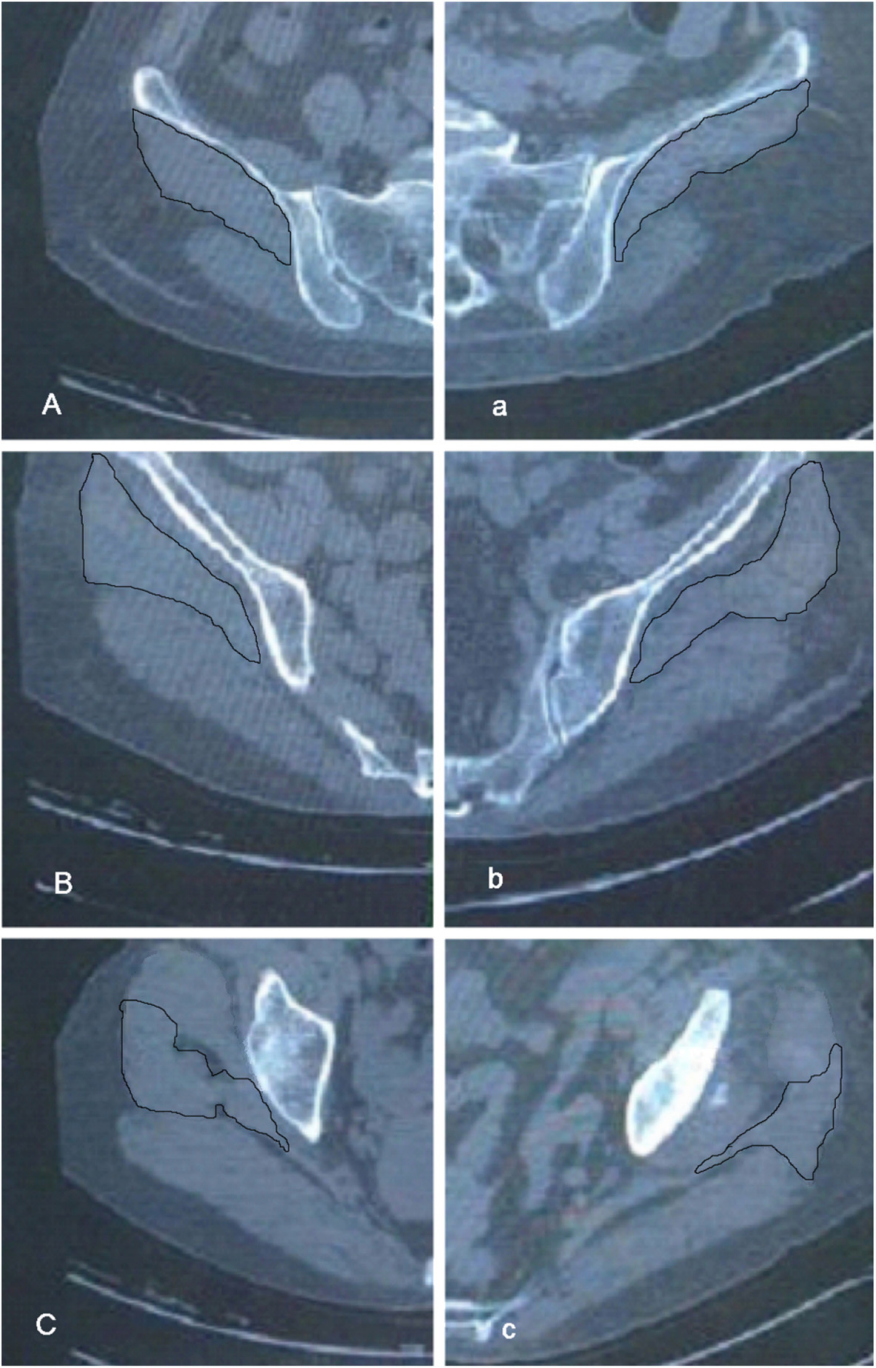
**Three different levels at which the Gluteus medius muscle was sectioned.** Sections **A–C**, arrows indicate gluteus medius muscle on the healthy side, which were compared to the smaller and less signal-intense muscles of the affected one in planes **a-c.**

Muscle cross-sectional area (CSA) and radiological density (RD; in Hounsfield units: HU) were assessed in three different level section of the gluteus medius muscle using transaxial CT scans. For accuracy, we selected three different sections levels of the muscle. We drew a line tangental to the lateral margin of the greater trochanter and extended the line to intersect with a horizontal line under the fifth lumbar which is approximately the gluteus medius origin plane. This line is also the gluteus medius line of action for the force. We quartered the line and chose three points (except the origin and end), through the points we drew a horizontal line, the plane corresponding to the spiral transaxial CT scans (Figure 1B). Areas of interest (the section of the gluteus medius muscle) were manually circumscribed and automatically computed (Figure 2A, B and C). The density was measured in Hounsfield units (HU) and was evaluated by measuring the mean density of the cross-sectional area in the region of interest, CSA and RD for gluteus medius muscle were determined twice by 2 independent observers to calculate the intra- and inter-observer reproducibility.

**Figure 3:**
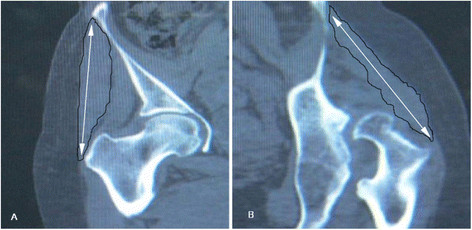
**Length of gluteus medius muscle.** Images indicate the difference of the length of gluteus medius muscle between the DDH hip**(B)** and healthy hip **(A)**. The variables were measured using the calibrated measuring tool of the computer work station (Phillips MX LiteView1.0, Phillips/Marconi Marconi Medical Systems Inc., Cleveland, Ohio, USA).

With the technique of multiplanar reconstruction (MPR), the frontal pelvic plane was established using both anterior superior iliac spines and the pubic tubercles and subsequent axes from this [19,25], we selected the frontal plane through greater trochanter, The length of the gluteus medius from its origin to its insertion was measured as a straight line [10] (shown in Figure 3).

Measurement of the parameters for the gluteus medius muscle were performed twice by 2 independent investigators, the first examiner performed the measurements twice (2 weeks apart), and the other performed it once. Two independent examiners analyzed the blinded images on two separate occasions at least 3 weeks apart. Intra-observer and inter-observer variations in measuring all parameters were assessed using the coefficient of variation (CV%).

### Statistics

Comparisons between the affected hip and the healthy side were performed using paired t-tests. Statistical significance was set at p < 0.05, for repeated tests. All data analysis was performed with SPSS 13.0 (SPSS, Chicago, Ill., USA).

## Results

The intra-observer and inter-observer repeatability of measurements was acceptable. Intra-observer reproducibility (CV%) for parameters measurements ranged from 0.7 % to 3.5 % and inter-observer reproducibility varied from 2.1 % and 7.8 %.

The cross-sectional area (CSA) of the gluteus medius muscle demonstrated no statistical difference in the A(a) plane but was significantly decreased in the B(b) and C(c) plane in the affected hip compared to the control side (p < 0.05). The RD of the gluteus medius muscle decreased in the affected side compared with the control side (p < 0.05). The CSA of the gluteus medius muscle was reduced by17–22 % in the affected hip, whereas the RD of the muscle groups was reduced by 32-38 % relative to the control side at three planes (shown in Table 1 and Figure 2).

**Table 1:** Cross sectional area and radiological density characteristics

	Affected side	Healthy side	Difference (%)	P-value
**cross-sectional area (cm**^**2**^**)**				
A(a) plane	14.8(4.1)	17.9(5.5)	−17.3	0.06
B(b) plane	18.9(5.7)	23.6(6.4)	−19.9	0.001
C(c) plane	13.5(4.7)	17.4(5.1)	−22.4	0.005
**Density of muscle (HU)**				
A(a) plane	32.1(10.2)	49.3(6.9)	−34.9	0.002
B(b) plane	28.5(5.8)	46.5(8.1)	−38.7	0.001
C(c)plane	31.3(8.3)	46.2(6.8)	−32.3	0.001

Mean value(SD),percentage difference and p-value for cross-sectional area and radiological density in healthy compared to affected limbs in 19 patients with unilateral DDH.

A,B,C: the healthy side.

a,b,c: the affected side.

(−)means reduction compared to healthy side.

HU: houndsfield units.

The femoral neck-shaft angle significantly increased (p < 0.05) in the affected hip accompanied by a significant decrease in the femoral offset (p < 0.05). The length of the gluteus medius muscle was reduced by 8-11 % (p < 0.05) but the gluteus medius activation angle was significantly increased (p < 0.05) and hip abductor moment arm decreased (p < 0.05) in the affected side compared with the control side.

There was no significant difference in the height of the center of femoral head between the affected side and the control although it had slightly changed between the two sides (shown in Table 2).

**Table 2:** **hip abductor function related parameters in** 19 **patients wit**h **unilateral DDH**

	**Affected side** Mean value(SD)	**Healthy side** Mean value(SD)	**Difference (%)**	**P-value**
**Femoral neck-shaft angle (°)**	136.2(3.1)	126.1(4.5)	8	0.001
**Femoral offset (mm)**	27.23(3.5)	39.9(6.5)	−31.8	0.001
**Height of the center of femoral head (mm)**	52(8.7)	52.2(5.5)	−0.3	0.940
**Hip abductor moment arm (mm)**	34.89(6.5)	44.8(7.9)	−22.3	0.010
**The length of gluteus medius muscle (mm)**	135.6(10.3)	145.5(9.6)	−6.8	0.001
**The angle of gluteus medius activation(°)**	26.9(5.8)	12.4(2.8)	116.8	0.002

Mean value(SD), percentage difference and p-value for femoral offset, femoral neck-shaft angle, hip abductor moment arm and the angle of gluteus medius activation in healthy compared to affected limbs in 19 patients with unilateral DDH.

(−)means reduction compared to healthy side.

## Discussion

Our study demonstrates that the gluteus medius muscle shows marked atrophy in the affected hip. This phenomenon was also shown in subjects with advanced OA hip joint pathology. Gait patterns may provide some further explanation for the gluteus medius muscle response to advanced OA hip joint pathology [26,27]. The gluteus medius muscle atrophy appears inherently linked to offloading strategies used in gait during late stage joint pathology [28]. However, the cause in patients with DDH may be related not only with these factors described above but also the femur being displaced proximally, the femur being in a position of abduction with the reduced abductor lever arm and increased gluteus medius activation angle.

In contrast to other studies which selected only one plane both in the affected side and normal side [17,18],we choose three different sections. This approach can help to obtain more information about the gluteus medius muscle. We selected different planes in the contralateral healthy hip and the affected one taking into consideration the proximal migration of the femur on the dysplastic side. We selected the contralateral healthy hip as the control group maintaining consistency across patients despite the differing effects of age and height between the patient population we examined, which in itself can affect muscle quality and quantity.

The gluteus medius muscle is one of the main soft tissues that limit femur reduction and limb length discrepancy correction in patients with DDH. To overcome this problem, the release of the gluteus medius muscle is necessary in the mildly dysplastic hip [14] and combined with femoral osteotomies in the severely dysplastic hip [12]. Except one-step soft tissue releases, slow release by continuous iliofemoral distraction were also described [29,30] in the severely dysplastic hip. Either technique will affect the overall length and function of the gluteus medius muscle. Therefore, how much the gluteus medius muscle could be released and lengthened needs to be further evaluated. The contracture extent of soft tissue around the hip was thought to be an important factor for limb elongation length [31] in patients with DDH.

Accompanied with the contracuture of abductor musculature, the adductor musculature also does contract. Thus, except the gluteus medius muscle release, proper release of adductor muscles is vital to keep abduction-adduction balance and hip stability during hip reconstruction in THA.

Patient age at the time of the operation and the postoperative changes in the volume of the gluteus medius muscle are related to abductor muscle strength restoration in pelvic support osteotomy of the congenital hip dislocation [11]. The preoperative muscle strength was an essential factor in postoperative muscle strength recovery in patients with DDH, for whom abductor-sparing periacetabular Osteotomy was performed [32]. The posterolateral approach, which has a lesser disruption of abductor musculature and more anatomic dissection [33], should be adopt in patients with DDH performed THA.

Muscle strength is proportional to not only the muscle volume or cross-sectional area (CSA) but also the muscle radiological density (RD), which may represent the actual amount of contractile muscle [34]. Thus we used CSA combined with muscular RD [17,35,36] to evaluate the atrophy of gluteus medius muscle. The reduced CSA and RD of gluteus medius muscle in patients with DDH implicated reduced muscle strength. Trendelenburg sign, which is indicated by gluteus medius muscle weakness, often could not be improved in some patients performed THA [12-14]. The reduced muscle strength may be one of the causes of abductor dysfunction. At the same time, the abnormal femoral offset and abductor moment arm should be paid attention to in abduction function reconstruction in patients with DDH during THA, because the abductor function deficit may result from an intrinsically reduced muscular strength or may be the indirect result of biomechanical alterations induced by abnormal femoral offset and abductor moment arm.

The gluteus medius muscle has the reduced strength preoperative and length changes postoperative in patients with DDH during THA, Some specific rehabilitation exercise should be designed to strengthen the muscle and keep the stability of hip post-operative as for hip arthroscopy post-operative [37]. Weight-bearing exercises may provide more functional benefit because this type of exercise often activates a greater number of muscle group [38]. Muscle strength recovery and gait adaption was not complete one year after total hip arthroplasty in patients with unilateral osteoarthritis or osteonecrotic hips [39,40]. Another study demonstrated a slow morphological recovery in cross-sectional area (CSA) and radiological density (RD) of hip muscles compared to the healthy limb two years after THA in patients with osteoarthritis [41]. Considering the changes of gluteus medius muscle in patients with DDH, we think that patients need more time for muscle strength recovery and gait adaption.

## Conclusions

In conclusion, the gluteus medius muscles showed a substantial loss of CSA and RD, as well as length decrease in adult patients with unilateral DDH. The factors which influence the gluteus medius muscle were the abductor lever arm and gluteus medius muscle activation angle changes. Attention should be paid to these changes in hip reconstruction. These findings provide orthopedic surgeons with objective information about the amount and condition of the gluteus medius muscle in patients with DDH.

## Competing interests

The authors declared that they have no competing interest.

## Authors’ contributions

RYL and KZW designed the study. XDW contributed to the measurements and ZQT wrote the draft of the manuscript. CSW performed the statistical analyses and all authors read and approved the final version.

## Pre-publication history

The pre-publication history for this paper can be accessed here:

http://www.biomedcentral.com/1471-2474/13/101/prepub

## References

[B1] Pfirrmann CW Notzli HP Dora C Hodler J Zanetti M Abductor tendons and muscles assessed at MR imaging after total hip arthroplasty in asymptomatic and symptomatic patients Radiology 2005 235 3 969 976 10.1148/radiol.2353040403 15860673

[B2] Amaro AJ Amado F Mendes A Oliveira J Malheiro A Meireles A Appell HJ Duarte JA Radiographic geometric measures of the hip joint and abductor muscle function in patients after total hip replacement Eur J Orthop Surg Traumatol 2007 17 437 443 10.1007/s00590-007-0207-3

[B3] Asayama I Chamnongkich S Simpson KJ Kinsey TL Mahoney OM Reconstructed Hip Joint Position and Abductor Muscle Strength After Total Hip Arthroplasty J Arthroplasty 2005 20 4 414 420 10.1016/j.arth.2004.01.016 16124955

[B4] Kiyama T Naito M Shitama H Maeyama A Effect of Superior Placement of the Hip Center on Abductor Muscle Strength in Total Hip Arthroplasty J Arthroplasty 2009 24 2 240 245 10.1016/j.arth.2008.08.012 18835515

[B5] Preininger B Schmorl K von Roth P Winkler T Schlattmann P Matziolis G Perka C Tohtz S A formula to predict patients' gluteus medius muscle volume from hip joint geometry Man Ther 2011 16 5 447 451 10.1016/j.math.2011.02.003 21414832

[B6] Kumagai M Shiba N Higuchi F Nishimura H Inoue A Functional evaluation of hip abductor muscles with use of magnetic resonance imaging J Orthop Res 1997 15 6 888 893 10.1002/jor.1100150615 9497815

[B7] Lai KA Lin CJ Su FC Gait analysis of adult patients with complete congenital dislocation of the hip J Formos Med Assoc 1997 96 740 744 9308329

[B8] Sanchez-Sotelo J Berry DJ Trousdale RT Cabanela ME Surgical treatment of developmental dysplasia of the hip in adults: II. Arthroplasty options J Am Acad Orthop Surg 2002 10 5 334 344 12374484 10.5435/00124635-200209000-00005

[B9] Wu X Lou LM Li SH Wu WP Cai ZD Soft tissue balancing in total hip arthroplasty for patients with adult dysplasia of the hip Orthop Surg 2009 1 3 212 215 10.1111/j.1757-7861.2009.00034.x 22009845 PMC6583671

[B10] Inan M Alkan A Harma A Ertem K Evaluation of the gluteus medius muscle after a pelvic support osteotomy to treat congenital dislocation of the hip J Bone Joint Surg Am 2005 87 10 2246 2252 10.2106/JBJS.D.02727 16203890

[B11] Inan M Mahar A Swimmer T Tomlinson T Wenger DR Changes in the lengths of the gluteus medius and gluteus minimus muscles with trochanteric transfer following pelvic support osteotomy, a biomechanical study Acta Orthop Traumatol Turc 2004 38 1 67 70 15054301

[B12] Togrul E Ozkan C Kalaci A Gülşen M A new technique of subtrochanteric shortening in total hip replacement for Crowe type 3 to 4 dysplasia of the hip J Arthroplasty 2010 25 3 465 470 10.1016/j.arth.2009.02.023 19577893

[B13] Reikerås O Haaland JE Lereim P Femoral Shortening in Total Hip Arthroplasty for High Developmental Dysplasia of the Hip Clin Orthop Relat Res 2010 468 7 1949 1955 10.1007/s11999-009-1218-7 20077043 PMC2881990

[B14] Hartofilakidis G Karachalios T Total hip arthroplasty for congenital hip disease J Bone Joint Surg Am 2004 86A 2 242 250 14960667 10.2106/00004623-200402000-00005

[B15] Bouche KG Vanovermeire O Stevens VK Coorevits PL Caemaert JJ Cambier DC Verstraete K Vanderstraeten GG Danneels LA Computed tomographic analysis of the quality of trunk muscles in asymptomatic and symptomatic lumbar discectomy patients BMC Musculoskelet Disord 2011 Mar 31 12 65 10.1186/1471-2474-12-65 21453531 PMC3079706

[B16] Clark JM Haynor DR Anatomy of the abductor muscles of the hip as studied by computed tomography J Bone Joint Surg Am 1987 69 7 1021 1031 3654693

[B17] Rasch A Byström AH Dalen N Berg HE Reduced muscle radiological density, cross-sectional area, and strength of major hip and knee muscles in 22 patients with hip osteoarthritis Acta Orthop 2007 78 4 505 510 10.1080/17453670710014158 17966005

[B18] Preininger B Schmorl K von Roth P Winkler T Matziolis G Perka C Tohtz S The sex specificity of hip-joint muscles offers an explanation for better results in men after total hip arthroplasty Int Orthop 2012 36 6 1143 1148 10.1007/s00264-011-1411-7 22134706 PMC3353095

[B19] Liu RY Wang KZ Wang CS Dang XQ Tong ZQ Evaluation of Medial Acetabular Wall Bone Stock in Patients with Developmental Dysplasia of the Hip Using a Helical Computed Tomography Multiplanar Reconstruction Technique Acta Radiol 2009 50 791 797 10.1080/02841850903049366 19629771

[B20] Crowe JF Mani VJ Ranawat CS Total hip replacement incongenital dislocation and dysplasia of the hip J Bone Joint Surg Am 1979 61 1 15 23 365863

[B21] McGrory BJ Morrey BF Cahalan TD An KN Cabanela ME Effect of femoral offset on range of motion and abductor muscle strength after total hip arthroplasty J Bone Joint Surg Br 1995 77 865 869 7593096

[B22] Hoaglund FT Low WD Anatomy of the femoral neck and head, with compara-tive data from Caucasians and Hong Kong Chinese Clin Orthop Relat Res 1980 152 10 16 7438592

[B23] Sakai T Sugano N Ohzono K Nishii T Haraguchi K Yoshikawa H Femoral anteversion, femoral offset, and abductor lever arm after total hip arthroplasty using a modular femoral neck system J Orthop Sci 2002 7 1 62 67 10.1007/s776-002-8418-7 11819134

[B24] Lecerf G Fessy MH Philippot R Massin P Giraud F Flecher X Girard J Mertl P Marchetti E Stindel E Femoral offset: Anatomical concept, definition, assessment, implications for preoperative templating and hip arthroplasty Orthop Traumatol Surg Res 2009 95 3 210 219 10.1016/j.otsr.2009.03.010 19423418

[B25] Mayr E Kessler O Prassl A Rachbauer F Krismer M Nogler M The frontal pelvic plane provides a valid reference system for implantation of the acetabular cup:spatial orientation of the pelvis in different positions Acta Orthop 2005 76 848 853 10.1080/17453670510045471 16470440

[B26] Arokoski MH Arokoski JP Haara M Kankaanpaa M Vesterinen M Hip muscle strength and muscle cross sectional area in men with and without hip osteoarthritis J Rheumatol 2002 29 10 2187 2195 12375331

[B27] Grimaldi A Richardson C Stanton W Durbridge G Donnelly W Hides J The association between degenerative hip joint pathology and size of the gluteus medius, gluteus minimus and piriformis muscle Man Ther 2009 14 6 605 610 10.1016/j.math.2009.07.004 19695944

[B28] Krebs DE Robbins CE Lavine L Mann RW Hip biomechanics during gait.Journal of Orthopedic and Sports Phys Ther 1998 28 1 51 59 10.2519/jospt.1998.28.1.51 9653690

[B29] Lerch M Thorey F von Lewinski G Klages P Wirth CJ Windhagen H An alternative treatment method to restore limb-length discrepancy in osteoarthritis with high congenital hip dislocation Arch Orthop Trauma Surg 2009 129 12 1593 1599 10.1007/s00402-009-0846-4 19271228

[B30] Lai KA Shen WJ Huang LW Chen MY Cementless total hip arthroplasty and limb-length equalization in patients with unilateral Crowe type-IV hip dislocation J Bone Joint Surg Am 2005 87 339 345 10.2106/JBJS.D.02097 15687157

[B31] Fujishiro T Nishiyama T Hayashi S Kurosaka M Kanno T Masuda T Leg Length Change in Total Hip Arthroplasty With Subtrochanteric Femoral Shortening Osteotomy for Crowe Type IV Developmental Hip Dysplasia J Arthroplasty 2012 27 6 1019 1022 10.1016/j.arth.2012.01.032 22480527

[B32] Ezoe M Naito M Asayama I Muscle strength improves after abductor-sparing periacetabular osteotomy Clin Orthop Relat Res 2006 444 161 168 16449917 10.1097/01.blo.0000196475.40151.8b

[B33] Padgett DE Warashina H The unstable total hip replacement Clin Orthop Relat Res 2004 420 72 79 Review 15057081 10.1097/00003086-200403000-00011

[B34] Goodpaster BH Carlson CL Visser M Kelley DE Scherzinger A Harris TB Stamm E Newman AB Attenuation of skeletal muscle and strength in the elderly: The health abc study J Appl Physiol 2001 90 6 2157 2165 11356778 10.1152/jappl.2001.90.6.2157

[B35] Hu ZJ He J Zhao FD Fang XQ Zhou LN Fan SW An Assessment of the Intra- and Inter-reliability of the Lumbar Paraspinal Muscle Parameters UsingCT Scan and Magnetic Resonance Imaging Spine 2011 36 13 868 874 Phila Pa 1976 10.1097/BRS.0b013e3181ef6b51 21224757

[B36] Keller A Brox JI Gunderson R Holm I Friss A Reikerås O Trunk Muscle Strength, Cross-sectional Area, and Density in Patients With Chronic Low Back Pain Randomized to Lumbar Fusion or Cognitive Intervention and Exercises Spine 2004 29 1 3 8 Phila Pa 1976 10.1097/01.BRS.0000103946.26548.EB 14699268

[B37] Philippon MJ Decker MJ Giphart JE Torry MR Wahoff MS LaPrade RF Rehabilitation exercise progression for the gluteus medius muscle with consideration for iliopsoas tendinitis: an in vivo electromyography study Am J Sports Med 2011 39 8 1777 1785 10.1177/0363546511406848 21566069

[B38] Jacobs CA Lewis M Bolgla LA Christensen CP Nitz AJ Uhl TL Electromyographic Analysis of Hip Abductor Exercises Performed by a Sample of Total Hip Arthroplasty Patients J Arthroplasty 2009 24 7 1130 1136 10.1016/j.arth.2008.06.034 18757169

[B39] Shih CH Du YK Lin YH Wu CC Muscular Recovery Around the Hip Joint After Total Hip Arthroplasty Clin Orthop Relat Res 1994 302 115 120 8168288

[B40] Foucher KC Hurwitz DE Wimmer MA Preoperative gait adaptations persist one year after surgery in clinically well-functioning total hip replacement patients J Biomech 2007 40 15 3432 3437 10.1016/j.jbiomech.2007.05.020 17644101

[B41] Rasch A Bystrom AH Dalen N Martinez-Carranza N Berg HE Persisting muscle atrophy two years after replacement of the hip J Bone Joint Surg Br 2009 91 583 588 10.1302/0301-620X.91B5.21477 19407289

